# Cardiovascular disease in type 2 diabetes mellitus: progress toward personalized management

**DOI:** 10.1186/s12933-022-01516-6

**Published:** 2022-05-14

**Authors:** Cheng-Xu Ma, Xiao-Ni Ma, Cong-Hui Guan, Ying-Dong Li, Dídac Mauricio, Song-Bo Fu

**Affiliations:** 1 Department of Endocrinology, The First Hospital of Lanzhou University, No. 1 West Donggang Road, Lanzhou, Gansu 730000 People’s Republic of China; 2 The First Clinical Medical College of Lanzhou University, Lanzhou, 730000 Gansu China; 3 College of Integrated Traditional Chinese and Western Medicine, Gansu University of Chinese Medicine, Lanzhou, 730000 Gansu China; 4 Department of Endocrinology & Nutrition, CIBER of Diabetes and Associated Metabolic Diseases (CIBERDEM), Hospital de la Santa Creu i Sant Pau, Autonomous University of Barcelona, 08041 Barcelona, Spain

**Keywords:** Type 2 diabetes mellitus, Cardiovascular diseases, Hyperlipidemia, Hypertension, Therapy principle, Antidiabetic therapy

## Abstract

Cardiovascular diseases (CVDs) are the main cause of death among patients with type 2 diabetes mellitus (T2DM), particularly in low- and middle-income countries. To effectively prevent the development of CVDs in T2DM, considerable effort has been made to explore novel preventive approaches, individualized glycemic control and cardiovascular risk management (strict blood pressure and lipid control), together with recently developed glucose-lowering agents and lipid-lowering drugs. This review mainly addresses the important issues affecting the choice of antidiabetic agents and lipid, blood pressure and antiplatelet treatments considering the cardiovascular status of the patient. Finally, we also discuss the changes in therapy principles underlying CVDs in T2DM.

## Introduction

Type 2 diabetes mellitus (T2DM) is a persistent state of hyperglycemia and glucose intolerance that occurs when the body cannot respond fully to insulin, followed by an increase in insulin production and a subsequent insulin deficiency. Once mainly limited to older adults in the twentieth century, T2DM is now the largest global health crisis of the twenty-first century, and although its prevalence and incidence have shown the fastest increase in adults, T2DM is increasingly occurring in children and adolescents [1]. Premature mortality from diabetes increased by 5% during the period 2000–2016 [2]. Between 2000 and 2019, trends in deaths due to diabetes exhibited a 3% increase in age-standardized rates [3]. Of note, T2DM accounts for approximately 90% of all diabetes cases, and cardiovascular (CV) events in people with T2DM are a major cause of the increased risk of early death and have become a rising threat to human health worldwide.

The major CV diseases (CVDs) associated with T2DM include ischemic heart disease, heart failure (HF), stroke, coronary artery disease (CAD), and peripheral artery disease, and these complications can result in death for at least 50% of patients with T2DM [4]. Therefore, CVDs are of great concern in the disease progression and prognosis of T2DM. T2DM is characterized by insulin resistance and hyperglycemia, which is usually, but not always, accompanied by abnormal lipid metabolism. Insulin resistance generally occurs early in the progression of T2DM and CVD [5]. Importantly, insulin resistance is associated with a higher relative risk of CV events [6]. Elevated blood glucose is strongly associated with the risk of both macrovascular and microvascular complications in patients with T2DM [7]. Furthermore, excess accumulation of lipids may result in cardiac insulin resistance, fibrosis and diastolic dysfunction [8]. Moreover, hypoglycemia, which has generally been recognized as an adverse effect of glucose reduction, has also been reported as a risk factor for CVD among patients with T2DM [9]. Overall, CVD associated with diabetes is a major cause of death and disability among patients with T2DM.

To date, limited progress has been made in the prevention of T2DM, and no country or health care system is immune to the threat of T2DM. Although the incidence and mortality rate of CVD have decreased in high-income countries, these countries account for only approximately 10% of the world population, and CVD incidence and mortality trends in T2DM patients in middle- and low-income countries remains unclear [10]. In addition, considering the clinical burden of CVD complications in T2DM patients, attention to the joint management of T2DM and CVD has been increasing. In this review, we focus more thoroughly on the current state of the epidemiology of CVD in T2DM and on preventive measures and then explore measures to reduce the threat from CVD in T2DM patients.

## Current trends in the epidemiology of cardiovascular disease in T2DM

While globally the incidence of CVD among patients with T2DM is 2–3 times higher than among those without T2DM, data suggest a decreasing trend worldwide in the overall prevalence of CVD attributed to T2DM. The currently available data are shown in Table 1. Before 2016, the prevalence of all CVDs in T2DM ranged from 14.3 to 46.9%[11], while a meta-analysis for the years 2007–2017 indicated a prevalence of 32%[4]. The weighted CVD prevalence in 2019 was reported to be 34.8% across 13 countries, although there was a wide range between countries from 18.0% in Saudi Arabia to 56.5% in Israel. [12]. There was a general trend of reduced incidence of coronary artery disease, myocardial infarction, stroke and HF, and peripheral artery disease [12]. The study by Artime et.al demonstrated that the prevalence of CVD among patients with T2DM in Spain was ranged from 7 to 41%, and in-hospital mortality rates due to CVD were between 6 and 11%[13].

**Table 1:** Prevalence of cardiovascular disease in T2DM

Outcome	Before 2016 [11]	2007–2017 [4]	2019 [12]
All CVDs	14.3–46.9%	32%	34.8%
Coronary artery disease	1.8–25.6%	21%	10.9%
Heart attack	3.3–17.8%	10%	4.6%
Stroke	1.7–17.7%	7.6%	5%
Heart failure		14.9%	2.4%
Peripheral artery disease			2.6%

*CVD* cardiovascular disease

The reduction in the incidence of CVD in T2DM is consistent with new findings from Ethiopia [14], Sweden [15], and South Korea [16]. However, in the study from South Korea the risk of HF increased over the years 2006 to 2015 [16], and this finding was also observed among patients with a T2DM duration over 10 years and among those with a history of hypertension [17]. The occurrence of HF has been found to be related to diabetes duration, with a 17% increase in HF risk strongly associated with each 5-year increase in diabetes duration [18].

The decreasing incidence of CVD among T2DM patients has been reported in high-income countries, such as Sweden, the USA, Canada, and the United Kingdom, with a 3–5% yearly decline in the rates of CVD since the early 1990s [19]. However, a large gap remains in the incidence of CV morbidity and mortality between people with T2DM and those without the disease. Unfortunately, there is a paucity of data on CVD incidence among people with T2DM from middle- and low-income countries and in global investigations.

Consistent with the declining trend of CVD incidence, the incidence of death from CVD in T2DM steadily dropped in 2019 [3]. There was approximately a 20% reduction in the mortality rates in Sweden from 1998 through 2014 [15]. Additionally, a general trend toward a reduction in T2DM mortality was reported in countries from the European Union between 1990 and 2019 [20]. Likewise, reductions in the death rate and the proportion of deaths from vascular causes decreased from 1988–94 to 2010–15 in the USA [21]. A similar trend was also observed in Hong Kong [22] and South Korea [16]. However, in contrast to the findings of observational studies, a systematic review of diabetes CV outcome clinical trials reported no improvement in CV mortality in T2DM; the possible cause underlying the difference between randomized controlled trials (RCTs) and real world data may be the lack of standardized definitions of CVD outcomes (including HF) [23]. The overall estimated CVD mortality rates among patients with diabetes are higher in low- and middle-income countries than in high-income countries [24]. Thus, the global CVD-related mortality among people with diabetes in low-income countries may decrease in time, in-line with high-income countries.

Despite the trend in the reduction in the overall CVD mortality, the incidence and mortality rate of T2DM continue to increase annually, and along with population growth and aging, the total number of CVD- and T2DM-related deaths have increased worldwide [3]. The highest number of deaths due to diabetes in adults in 2019 occurred in the Western Pacific region, followed by Southeast Asia, and the highest proportion of deaths due to diabetes among patients under the age of 60 in 2019 was in Africa [24]. CVD and diabetes were among the top ten causes of death globally in 2019 and are a considerable threat to human health.

A transition in the major cause of death in T2DM from CVD to non-CVD was observed in the ALTITUDE trial [25]. Non-vascular, non-cancer causes of death among American adults diagnosed with diabetes accounted for approximately 46.5% of all deaths during 2010–2015, and the proportion of cancer-related deaths did not change from 1988 to 2015 [21]. A change in the leading cause of death from vascular causes to cancer among people with diabetes was observed in England from 2001 to 2018 [26]. In addition, a decrease in mortality due to cancer and CVD in older people with T2DM in Australia was observed, masking the rising excess risk among patients of younger ages [27]. There is a suggestion that younger-onset T2DM may be associated with an increase in CVD-related mortality but a slight decrease in cancer-related mortality [28]. In summary, notwithstanding some reductions in CVD mortality in T2DM, there is a paucity of data about the leading cause of death among T2DM patients, especially at younger ages.

## Prevention of cardiovascular disease in T2DM

A focus on efforts to prevent CVD events in high-risk populations might reduce mortality and decrease the economic burden of heart attack and stroke. CVD can be prevented or delayed by controlling blood glucose, blood pressure and cholesterol and with lifestyle changes such as stopping smoking, eating healthily, and increasing physical activity.

Primary prevention involves preventing or delaying new-onset CVD in patients with T2DM. Early detection of CVD-related risk factors in community-based high-risk populations and early diagnosis of T2DM in subjects without CVD are needed. Secondary prevention involves treatment of risk factors in diabetic patients with established CVD. Comprehensive medical intervention for secondary prevention among diabetic patients with clinical CVD was approved by the American Heart Association (AHA) in 1999 [29]; a position statement by the American Diabetes Association (ADA) and the European Association for the Study of Diabetes (EASD) in 2012 outlined the need for individualized glycemic targets and glucose-lowering therapies in combination with comprehensive CV risk reduction, indicating that patient-centered care must be a consideration in decision-making for secondary prevention among T2DM patients with established CVD [30]. Beginning in 2018, the glucocentric strategy for the management of CVD in T2DM transitioned to patient-centered care [31], and individual medical history, lifestyle behaviors, and CV and metabolic risk factors were included as considerations. Precision medical therapeutic approaches for patients with T2DM with CVD will be gradually achieved [32].

CVD risk assessment aims to provide appropriate regimens for the prevention and treatment of CVD in T2DM. The American College of Cardiology (ACC) and AHA have developed a risk stratification tool for primary prevention in patients with T2DM; the Risk Estimator Plus tool can be used to calculate ten-year atherosclerosis CVD risk and provide individualized advice for patients aged 40–79 years. Risk stratification for secondary prevention is under clinical testing [33]. Alternatively, the European Society of Cardiology (ESC) recommends that CVD risk stratification be divided into moderate-, high-, and very high-risk levels for patients with either pre-DM or established DM based on comorbidities and the duration of the disease, rather than into primary or secondary prevention groups. These three levels of risk stratification support individualized diagnostic and therapeutic approaches to patient care in diabetes [34].

The ACC/AHA and ESC methods differ somewhat in the criteria they use for risk stratification. The Risk Estimator Plus does not account for the comorbidities and duration of diabetes, especially for adolescents due to the increase in obesity incidence, but it does consider age ≥ 40 years, sex, blood pressure, blood lipids, history of diabetes, smoking and drug use. Risk stratification for secondary prevention accounts for age ≥ 75 years, DM, hypertension, peripheral artery disease, previous stroke, previous coronary artery bypass graft, history of HF, active smoking, and renal dysfunction. The ESC method based on three levels of risk stratification recognizes the complexity of developing CVD by considering comorbidities and the duration of diabetes, and also accounts for obesity, renal impairment, left ventricular hypertrophy, and retinopathy rather than a history of CV-related disease. A comparison of the benefits of the risk estimator and that of risk stratification would be useful to obtain a consensus to facilitate the choice of individualized care in patients with T2DM and CVD.

## Individualized glycemic management in patients with T2DM and CVD

A meta-analysis of 102 prospective studies showed that fasting blood glucose concentrations ≥ 7 mmol/L conferred an elevated risk of coronary heart disease [35]. Additionally, impaired glucose tolerance and elevated HbA1c (> 5.7%) levels have been associated with an increased risk of CVD [36]. An increased blood glucose concentration in the general population or in patients with atherosclerotic CVD confers an increased risk of all-cause mortality and CVD [37]. Individualized glycemic management may lead to improved prevention of CVD in T2DM. In this section, we focus on current aspects of individualized management of T2DM.

## Glycemic targets

With respect to glycated hemoglobin (HbA1c) targets, the AHA, ADA, ESC and ESC/EASD guidelines propose a target of < 7%, and gives further recommendations for less stringent targets in older or more frail adults. A target of ≤ 6.5% for those with an early diagnosis, who are not frail, and who do not have established atherosclerotic CVD. This is consistent with current ESC, ADA and AHA guidelines on DM and CVD [38–41](Table 2).

**Table 2:** Targets for the control of glycemia

Parameter		Target	Population	Guideline	References
Glycemia	HbA1c	Consistent with current ADA guideline		AHA	[39]
		< 6%	Pregnancy patients without significant hypoglycemia	ADA	[38]
		< 6.5%	Children and adolescents patients with a short duration of DM, lesser degrees of β-cell dysfunction and no evidence of CVD		
		< 7%	Nonpregnant patients without significant hypoglycemia		
		< 8%	Patients with limited life expectancy or where the harms of treatment are greater than the benefits		
		< 7%	Patients with developing microvascular complications	ESC/EASD	[40]
		6.0–6.5%	Younger patients with a short duration of DM and no evidence of CVD		
		< 8% or ≤ 9%	Elderly patients with longstanding DM and limited life expectancy, and frailty with multiple comorbidities		
		< 7%	Older/frail adults	ESC	[41]
		≤ 6.5%	In early diagnosis, not frail and without established ASCVD		

*DM* diabetes Mellitus, *CVD* cardiovascular disease, *T2DM* type 2 diabetes mellitus, *ASCVD* atherosclerotic cardiovascular disease, *AHA* American Heart Association, *ADA* American Diabetes Association, *ESC* European Society of Cardiology, *EASD* European Association for the Study of Diabetes

Achieving a glycemic target of an HbA1c level < 7% for patients with T2DM reduces the incidence of the development of microvascular complications and CVD. A more stringent HbA1c goal (< 6.5%) is defined for selected children and adolescents, those with a short duration of DM, lesser degrees of β-cell dysfunction and no evidence of CVD. An HbA1c target of < 6% may be optimal during pregnancy without significant hypoglycemia. Furthermore, a less stringent HbA1c goal (< 8.0% or even slightly higher) may be appropriate for patients with limited life expectancy, a history of advanced microvascular or macrovascular complications, extensive comorbid conditions, or long-standing T2DM [38]. The association of HF mortality with HbA1c goals in T2DM has a “U” shape, where a modest HbA1c level of 7.1–8.0% is associated with the lowest mortality among patients with HF and T2DM [42], whereas an HbA1c level < 6.0% or > 8%, and even > 10%, may increase the risk of incident HF or HF-related hospitalization [43].

### Antidiabetic treatment choice

The novel glucose-lowering agents, sodium-glucose cotransporter 2 inhibitors and glucagon-like peptide-1 receptor agonists, have been shown to lower atherosclerotic CVD and HF risks independently of baseline HbA1c[44]; this has led to newer treatment approach. A reasonable regimen of glycemic control is associated with both glycemic targets and strategies of glucose-lowering therapy, and the medical provider must prioritize the patient’s CV state based on preference and the prescription history.

#### Metformin

The UK Prospective Diabetes Study (UKPDS 34) found that metformin therapy reduced all-cause mortality by 36% and heart attack incidence by 39% among overweight patients with newly diagnosed T2DM [45]. Moreover, a continued benefit of reductions in microvascular risk, myocardial infarction and death over 10 years after metformin therapy in that trial were reported [46]. Metformin administration may be the primary preventive measure against the risk of CVD among patients with newly diagnosed T2DM without established CVD. However, there is insufficient evidence for the use of metformin for secondary prevention in patients with T2DM and established CVD [47]. Only one RCT that included 68 patients with CAD showed a cardioprotective role of metformin [48], and in another trial, 36 patients with HF with reduced ejection fraction (HFrEF) showed increased myocardial efficiency after metformin treatment [49]. Nonetheless, the additional benefits of metformin may include prevention of the occurrence of new T2DM and cancer [50, 51], weight gain and hypoglycemia, and prolonged gestation in preterm preeclampsia [52]. Thus, metformin administration is suitable for the treatment of patients with T2DM without established CVD as well as those with CVD.

#### Sodium-glucose cotransporter 2 inhibitors

A meta-analysis of three studies showed that sodium-glucose cotransporter 2 (SGLT2) inhibitors reduced major adverse CV events by 11% in patients with T2DM and atherosclerotic CVD and reduced the risk of CV death or hospitalization for HF by 23% and the risk of renal disease progression by 45% among patients with T2DM [53]. SGLT2 inhibitors also reduced the risk of cardiac arrhythmias and atrial fibrillation/atrial flutter progression [54, 55].

Currently, the approved SGLT2 inhibitors are empagliflozin [56], canagliflozin [57], dapagliflozin [58], ertugliflozin [59], tofogliflozin [60], luseogliflozin [61] and ipragliflozin [61] (Table 3). The available studies focused on CVD are mainly limited to the United States and Europe; however, more recent real-world data have also shown a reduced risk of CV events across a wide range of patient characteristics from 6 countries in the Asia Pacific region, the Middle East, and North American regions [61]. In HF, empagliflozin administration was associated with improved clinical outcomes for the HFrEF phenotype [62], and the overall risk of HF events between HFrEF and HFpEF was reduced by canagliflozin [63], dapagliflozin [64], and ertugliflozin [65].

**Table 3:** Cardiovascular benefits of SGLT2 inhibitors

Agents	MACEs (HR)	CV death (HR)	HF (HR)	Renal outcome (HR)
Empagliflozin [56]	0.86	0.62	0.65	0.5
Canagliflozin [57]	0.86	0.87	0.67	0.6
Dapagliflozin [58]	0.93	0.98	0.73	0.76
Ertugliflozin [59]	0.97	0.92	0.7	0.81
Tofogliflozin [60]	–	–	–	–
Luseogliflozin [61]	–	–	–	–
Ipragliflozin [61]	–	–	–	–

– limited data, *MACE* major adverse cardiac event, *HR* hazard ratio, *CV* cardiovascular, *HF* heart failure

Overall, data suggest that SGLT2 inhibitors have moderate benefits in CVD and robust benefits in HF and renal disease and should be considered for primary and secondary prevention of CVD and renal outcomes in patients with T2DM, regardless of existing atherosclerotic CVD or a history of HF [53]. A recent meta-analysis by Giugliano et al. provided an update of all large CV outcome trials (CVOTs) with SGLT-2 inhibitors, with findings indicating that treatment with SGLT-2 inhibitors in patients with T2DM resulted in a sustained to moderate reduction of the composite CV death or hospitalization for HF, robust reduction of HF, moderate reduction of CV mortality, total mortality and major adverse cardiac events (MACE) [66]. Furthermore, a comprehensive systematic review and network meta-analysis of RCTs provided direct evidence for the absolute benefits and harms of SGLT-2 inhibitors in reducing CV outcomes in patients with type 2 diabetes [67]. Importantly, this comprehensive systematic review found that SGLT-2 inhibitors reduced all cause and CV mortality, myocardial infarction, admission for heart failure and kidney failure, without a reduction in non-fatal stroke.

In addition to the role of individual SGLT2 inhibitors, inhibitors of both SGLT1 and SGLT2 (dual SGLT1/2 inhibitors) have the ability to reduce intestinal glucose absorption after meals since SGLT1 in the small intestine [68]. Sotagliflozin, the first dual SGLT1/2 inhibitor, approved for T1DM and T2DM in the EU and T2DM in the US, has exhibited CV benefits in patients with T2DM and chronic kidney disease [69]; a similar result was shown in patients with T2DM and HFrEF or HFpEF [70]. The adverse events of sotagliflozin include diarrhea, genital mycotic infections, volume depletion, and diabetic ketoacidosis. Dual inhibition of SGLT1/2 with licogliflozin treatment showed CV benefits in patients with T2DM and HF, and licogliflozin has a greater effect than empagliflozin on glucose lowering, weight loss, and systolic blood pressure reduction [71]. The CV benefits of sotagliflozin seem to be equal to those of SGLT2 inhibitors. Sotagliflozin and licogliflozin may be recommended as first-line therapies among patients with T2DM, regardless of the presence of atherosclerotic CVD or a history of HF.

Other trials of SGLT1/2 inhibitors, including LX4211 [72], LX-2761, and YG-1699, for treating CVD in patients with T2DM are underway.

#### Glucagon-like peptide-1 receptor agonists

Five CVOTs, namely, LEADER3, SUSTAIN-6, REWIND, HARMONY, and AMPLITUDE-O [73] have consistently shown the safety and efficacy of GLP-1 agonists in patients with T2DM and have provided evidence of a secondary preventive effect of GLP-1 agonists in T2DM patients with CVD and kidney disease. Nevertheless, the ELIXA and EXSCEL trials did not show any benefit on CVD or kidney outcomes (Table 4). Currently, a notable decrease in CVD outcomes has been observed with several GLP-1 agonists; furthermore, GLP-1 agonists promote a reduction in CV risk factors, and dulaglutide showed strong evidence of primary prevention.

**Table 4:** Cardiovascular benefits of GLP-1 agonists

Trial	Agents	MACEs (HR/p)	CV death (HR/p)	HF (HR/p)	Renal outcome (HR/P)
ELIXA	Lixisenatide	1.02/0.78	0.98/0.85	0.96/0.75	0.84/0.083
LEADER3	Liraglutide	0.87/0.01	0.98/0.85	0.87/0.14	0.78/0.003
SUSTAIN-6	Semaglutide	0.74/0.016	0.98/0.92	1.11/0.57	0.64/0.005
EXSCEL	Exenatide	0.91/0.061	0.88/0.096	0.94/0.49	0.88/0.065
REWIND	Dulaglutide	0.88/0.026	0.91/0.21	0.93/0.46	0.85/0.0004
HARMONY	Albiglutide	0.78/0.0006	0.93/0.58	0.71/0.019	#
AMPLITUDE-O	Efpeglenatide	0.73/0.0069	0.72/0.07	0.61/0.04	0.68/ < 0.0001

*p* p values, *#* missing data, *MACE* major adverse cardiac events, *HR* hazard ratio, *CV* cardiovascular, *HF* heart failure

A systematic review and meta-analysis of RCTs revealed that GLP-1 receptor agonists reduced MACE by 14% and also reduced all-cause mortality by 12%, hospital admission for HF by 11%, and the composite kidney outcome by 21%, with no increase in risk of severe hypoglycemia, retinopathy, or pancreatic adverse effects [73]. Another meta-analysis showed that GLP-1 receptor agonists are efficacious for treating obesity and T2DM in children [74]. GLP-1 receptor agonists also safely reduce total MACEs by 12%, hospital admission for HF by 9%, and composite kidney outcomes (reduction in urinary albumin excretion) by 17% in adult T2DM patients [75]. Unexpectedly, a beneficial effect on HF has been observed with GLP-1 agonists. Furthermore, the largest and most current systematic review showed that GLP-1 agonists reduced all cause and CV mortality, myocardial infarction, non-fatal stroke, kidney failure and admission for HF in subjects with T2DM [67]. Overall, these important data provide novel insights into the benefits of GLP-1 receptor agonists in CVD patients; however, the benefits they may confer to primary prevention patients are unclear.

In summary, GLP-1 agonists show a beneficial effect on CVD, HF, and kidney outcomes and a reduction in weight gain, blood pressure, and levels of HbA1c and LDL-C. Therefore, GLP-1 agonists are recommended in the context of secondary prevention in patients with T2DM and CVD.

Finally, because the dual GIP/GLP-1 agonists will soon be introduced into clinical practice, it is worth addressing their potential. A phase 3 trial of tirzepatide showed that the mean HbA1c level decreased from baseline by 1.87–2.07%, more participants met their HbA1c targets, and weight loss ranged from 7.0 to 9.5 kg [76]. The efficacy of tirzepatide treatment in patients with T2DM was shown to be superior to that of treatment with 1.5 mg dulaglutide [77] and semaglutide [78]. Unfortunately, there are still no data on CVOTs with tirzepatide. However, tirzepatide treatment showed a good safety profile in T2DM patients with a high risk of or established CVD, an improved effect on HbA1c in comparison with insulin glargine, and no increases in MACE events compared to insulin glargine [79]. Available data show an important effect of tirzepatide in terms of HbA1c reduction, weight loss, blood pressure and lipid profiles, with this effect persisting for up to 2 years without increased CVD risk [80]. Tirzepatide displays primary preventive effects in T2DM with or without CVD risk and may be a well-positioned glucose-lowering agent in future therapeutic regimens.

Clinical trials of dual GIP/GLP-1 agonists, such as combined GIP and GLP-1 infusion [81] and NNC0090-2746 [82], are ongoing.

#### Other antidiabetic agents

Dipeptidylpeptidase-4 (DPP4) inhibitors have been reported to be neutral with regard to CV outcomes [83]. However, the use of saxagliptin is associated with an increase in hospitalization for HF [84], which should be carefully considered.

Regarding sulfonylureas, these older agents are inexpensive and widely available and are used as glucose-lowering agents for T2DM patients with CVD. The UKPDS 33 has confirmed the reduced microvascular risk of sulfonylureas [85]. In addition, the TOSCA.IT trial confirmed the CV safety of sulfonylureas [86]. Insulin is a widely extended therapeutic option. A meta-analysis showed that insulin treatment does not increase the risk of CV mortality and myocardial infarction [87]. A RCT showed that insulin glargine had a neutral effect on CV outcomes [88], and insulin is widely used to treat T2DM with CVD.

Finally, pioglitazone reduced key secondary macrovascular outcomes in people with T2DM without CV events whose glucose levels were controlled with metformin monotherapy [86]. Although the PROactive trial failed to show an effect of pioglitazone on the primary composite outcome, this drug resulted in a decrease in the secondary outcome (i.e., the classical 3 component MACE) [89]. Pioglitazone was also associated with a reduced risk of diabetes, ischemic stroke, and myocardial infarction in patients without diabetes [90]. An adverse effect of pioglitazone is an increased risk of developing HF, thus pioglitazone should not be used to treat subjects with HF. Therefore, pioglitazone may be used as an add-on treatment to metformin for people with T2DM, as these drugs are widely available and affordable.

## Current recommendations on the use of antidiabetic drugs with regard to CVD

Different scientific societies recommend the use of antidiabetic therapies in CVD, including the AHA [39], the ADA [91], the ESC [41] and the ESC/EASD [40]; Chinese scientific bodies, however, do not [92]. The overall medication regimen is roughly similar, with a key focus on metformin, GLP-1 agonists and SGLT2 inhibitors in guidelines for treating CVD in T2DM patients (Table 5). The ADA and the EASD issued an update of their joint 2018 recommendations on management of hyperglycemia [93]. The major updates included: 1) SGLT2 inhibitors are recommended in patients with T2DM and HF, particularly those with HFrEF, to reduce hHF, MACE, and CV death and to prevent the progression of CKD, HF, MACE, and CV death in patients with T2DM with CKD; 2) to reduce risk of MACE, GLP-1 receptor agonists can also be considered in patients with type 2 diabetes without established CVD with indicators of high risk, specifically, patients aged 55 years or older with coronary, carotid, or lower extremity artery stenosis > 50%, and left ventricular hypertrophy. 3) for patients with type 2 diabetes and established atherosclerotic CV disease (such as those with prior myocardial infarction, ischemic stroke, unstable angina with ECG changes, myocardial ischemia on imaging or stress test, or revascularization of coronary, carotid, or peripheral arteries) where MACE is the gravest threat, the level of evidence for MACE benefit is greatest for GLP-1 receptor agonists.

**Table 5:** Recommendations on the use of antidiabetic drugs

Antidiabetic drugs choice	Population	Guideline	References
SGLT-2 inhibitor and /or GLP-1 RA	Patients with T2DM with established atherosclerotic CVD, or multiple risk factors for CVD or established kidney disease irrespective of additional glucose lowering and metformin use	ADA	[91]
SGLT-2 inhibitor	Patients with T2DM and established HFrEF		
Initiated with metformin	Patients with T2DM and/or stable HF		
Initiated with an SGLT-2 inhibitor or GLP-1 RA	Patients with newly diagnosed T2DM and established atherosclerotic CVD, or very high risk factors for CVD before metformin use	ESC/EASD	[40]
Initiated with metformin	Overweight patients with T2DM without CVD and at moderate CV risk		
Metformin	In persons with T2DM with atherosclerotic CVD	ESC	[41]
Initiated with an SGLT-2 inhibitor or GLP-1 RA	In patients with T2DM without atherosclerotic CVD, HF, or chronic kidney disease		
SGLT-2 inhibitor, GLP-1 agonists and metformin	Consistent with current ADA guideline	AHA	[39]

*DM* diabetes mellitus, *CVD* cardiovascular disease, *T2DM* type 2 diabetes mellitus, *ASCVD* atherosclerotic cardiovascular disease, *ADA* American Diabetes Association, *ESC* European Society of Cardiology, *EASD* European Association for the Study of Diabetes, *HF* heart failure, *SGLT-2* sodium-glucose cotransporter -2, *GLP-1 RA* glucagon-like peptide-1 receptor agonist, *ACC* American College of Cardiology

The use of glucose-lowering agents should depend on CV status and T2DM complications. SGLT2 inhibitors (empagliflozin, canagliflozin, and dapagliflozin) are associated with a lower risk of HF hospitalization in patients with DM, and are recommended [94]. More importantly, the largest available systematic review found that SGLT2 inhibitors and GLP-1 receptor agonists lowered all-cause mortality, CV mortality, non-fatal myocardial infarction, and kidney failure. However, SGLT-2 inhibitors reduced admission to hospital for HF more than GLP-1 receptor agonists, and GLP-1 receptor agonists reduced non-fatal stroke more than SGLT-2 inhibitors (which appeared to have no effect) [67].

One RCT has shown the efficacy of metformin in patients with T2DM and HF [48]. Accordingly, metformin should be considered for treating patients with T2DM and HF (eGFR > 30 mL/min) [94]. Pioglitazone and saxagliptin are contraindicated in patients with HF or at risk of HF. Although initial data from RCTs of GLP-1 agonists supported a neutral effect on the risk of HF, a recent meta-analysis showed benefits of GLP-1 agonists in HF and diabetes [67]. However, current guidelines recommend SGLT2 inhibitors as the most suitable treatment in patients with HF.

## Hypertension

The presence of hypertension in patients with T2DM significantly increases the risk of CVD development, and a study revealed that evaluated blood pressure down to < 120 mm Hg and < 70 mm Hg could decrease mortality, macrovascular, and microvascular events regardless of baseline systolic blood pressure [39]. Thus, lowering blood pressure reduces CVD events and microvascular complications and has a favorable effect on CVD outcomes in patients with and without T2DM.

### Blood pressure treatment target

A target of 140/90 mmHg may be reasonable among patients with T2DM and stable CAD or patients with higher blood pressure targets; lower blood pressure targets of < 130/80 mmHg can be recommended for patients with a higher risk of stroke and microvascular complications, according to the AHA [95]. A target of < 130/80 mmHg may be appropriate for patients with diabetes and hypertension at higher CV risk (existing atherosclerotic cardiovascular disease (ASCVD) or 10-year ASCVD risk ≥ 15%), whereas a blood pressure target of < 140/90 mmHg is recommended for individuals with diabetes and hypertension at lower risk for CVD (10-year ASCVD risk < 15%). The ADA advises that a reading higher than 120/80 mmHg in patients with T2DM indicates the need for lifestyle intervention. In pregnant patients with diabetes and preexisting hypertension, a blood pressure target of 110–135/85 mmHg is suggested in the interest of reducing the risk for accelerated maternal hypertension [91]. ESC guidelines recommend that the initial target of < 140/90 mm Hg with goal of 120–130/ < 80 mm Hg is suitable for patients aged 18–69; the target of 130–139/ < 80 mm Hg, with < 130/ < 80 mm Hg being acceptable if tolerated is suitable for adults ≥ 70 years of age [41], although the blood pressure target for T2DM patients with CVD is unclear. Overall, individualized blood pressure targets are recommended based on CVD status and individual condition (Table 6).

**Table 6:** Targets for the control of blood pressure

Parameter	Target	Population	Guideline	References
Blood pressure	< 140/90 mmHg	Patients with T2DM and stable coronary artery disease or with higher blood pressure target	AHA	[95]
	< 130/80 mmHg	Patients with a higher risk of stroke and microvascular complications		
	< 140/90 mmHg	Patients with DM and hypertension at lower risk for CVD (10 year atherosclerotic CVD risk < 15%)	ADA	[91]
	< 130/80 mmHg	Patients with DM and hypertension at higher CV risk (existing atherosclerotic CVD or 10-year atherosclerotic CVD risk ≥ 15%)		
	110–135/85 mmHg	Pregnant women with DM and preexisting hypertension		
	< 120/80 mmHg	Patients who need lifestyle intervention		
	130—139 / < 80 but not < 70 mmHg	Older people (aged > 65 years)	ESC/EASD	[94]
	130/ < 80 but not < 70 mmHg and < 130/ < 80 but not < 70 mmHg if tolerated	Patients with hypertension and DM		
	< 140/90 mmHg	All treated patients	ESC	[41]
	120–130 / < 80 mmHg	Younger patients (18–69 years)		
	130–139/ < 80 mmHg and < 130 / < 80 mmHg if tolerated	Patients aged ≥ 70 years		

*DM* diabetes mellitus, *CVD* cardiovascular disease, *T2DM* type 2 diabetes mellitus, *ASCVD* atherosclerotic cardiovascular disease, *ADA* American Diabetes Association, *ESC* European Society of Cardiology, *EASD* European Association for the Study of Diabetes, *AHA* American Heart Association

### Treatment choice

The ADA and ESC guidelines consistently recommend that initial treatment should involve the drug classes demonstrated to reduce CVD events in patients with T2DM and hypertension, and dual therapy, ACE inhibitors or ARBs in conjunction with dihydropyridine calcium channel blockers or thiazide diuretics are recommended as the first-line treatment[41, 94]. For patients with a urine albumin-to-creatinine ratio ≥ 30 mg/g, initial treatment should include an ACE inhibitor or ARB. For patients with pre-DM, ACE inhibitors or ARBs rather than diuretics or β-blockers are recommended due to the increased risk of new-onset T2DM from diuretics and β-blockers [96]. Nebivolol does not reduce insulin sensitivity and may be used as an antihypertensive treatment for T2DM patients [97].

In addition, a double-blind clinical trial has shown that the non-steroidal potent MRA blocker finerenone is efficacious and safe to reduce HF cardiac biomarkers, HF, and albuminuria in T2DM. Different trials also demonstrated a significant reduction in the primary outcome (CV death, non-fatal myocardial infarction, non-fatal stroke, or hospitalization for HF) in those subjects with T2DM and chronic kidney disease receiving finerenone [98–100]. Indeed, finerenone significantly improved cardiorenal outcomes in patients with T2DM and kidney disease irrespective of HbA1c levels or insulin use [101]. Therefore, finerenone should be considered for treatment of patients with T2DM, and this agent is likely to be included as an important treatment option in future updates of current guidelines.

## Lipid management

The presence of diabetic dyslipidemia in patients with T2DM is, at least in part, a cause of CVD. Moreover, diabetic dyslipidemia, including elevated triglycerides, low-density lipoprotein cholesterol (LDL-C) and low high-density lipoprotein cholesterol (HDL-C), is associated with increased CV events, especially in high-risk populations [102]. Accounting for metabolic dyslipidemia in CVD risk stratification is necessary for patients with T2DM [103].

### Lipid targets

The concept of “lower is better” regarding LDL levels has been supported for reducing the risk of CVD in patients with T2DM and CVD [104]. The ADA guidelines recommend that lifestyle interventions be initiated for individuals with abnormal triglyceride levels (> 150 mg/dL) and/or HDL-C levels (< 40 mg/dL for men, 50 mg/dL for women), and an LDL-C target reduction of ≥ 50% or more from baseline is reasonable for patients with diabetes and 10-year ASCVD risk of 20% or higher [91]. The ESC/EASD guidelines recommend an LDL-C target of < 55 mg/dL for those with T2DM and at very high CV risk [94]. The AHA guidelines recommend an LDL-C target reduction of ≥ 50% from baseline for those with diabetes and clinical CVD [39]. The ESC established goals for LDL-C based on level of ASCVD risk [41]. With respect to lipid targets, the AHA/ADA and the ESC/EASD guideline are shown in Table 7.

**Table 7:** Targets for lipid control

Parameter		Target	Population	Guideline	References
Lipid	LDL-C	Reduction of ≥ 50% or more from baseline	Adults with diabetes and 10 year atherosclerotic CVD risk of 20% or higher	ADA	[91]
	LDL-C	Reduction of ≥ 50% from baseline	Patients with DM and atherosclerotic CVD	AHA	[39]
	LDL-C	Reduction of ≥ 50% from baseline and < 70 mg/dL	Patients with T2DM > 40 years of age at high CVD risk	ESC	[41]
		LDL-C Reduction of ≥ 50% from baseline and < 55 mg/dL	Patients with T2DM at very high CVD risk		
	LDL-C	< 100 mg/dL	Patients with T2DM at moderate CV risk	ESC/EASD	[94]
		< 70 mg/dL	Patients with T2DM at high CV risk		
		< 55 mg/dL	Patients with T2DM at very high CV risk		

*CV* cardiovascular, *ASCVD* atherosclerotic cardiovascular disease, *ADA* American Diabetes Association, *ESC* European Society of Cardiology, *EASD* European Association for the Study of Diabetes, *AHA* American Heart Association, *LDL-C* low-density lipoprotein cholesterol, *HDL-C* high-density lipoprotein cholesterol, *DM* diabetes mellitus

### Treatment choice

Meta-analysis has shown that statins are the most effective therapy for reducing CV mortality, followed by PCSK9 inhibitors and statins in combination with ezetimibe. PCSK9 inhibitors effectively reduce MACEs [105], and statin therapy and add-on treatment with PCSK9 inhibitors or ezetimibe exhibit significant benefit in CVD outcomes [106]. Fibrate therapy reduces major CVD events [107], and icosapent ethyl exerts a risk reduction in CVD outcomes beyond lipid-lowering effects [108]. Overall, these lipid-lowering drugs have CV benefits for secondary prevention.

Depending on the baseline LDL-C levels, the initiation of statin therapy remains the first-line treatment in patients (aged < 50 years) with a T2DM duration < 10 years and without CVD risk factors or with an LDL-C of > 100 mg/dL. In patients at very high CVD risk, if the LDL-C target is not reached, despite treatment with the maximum tolerated statin dose, combination therapy with ezetimibe or a PCSK9 inhibitor is recommended [41, 94]. In high- and very high-risk patients with triglycerides of 200–500 mg/dL, statins combined with a fibrate or icosapent ethyl may be considered for both macro- and microvascular benefits in patients with T2DM [109]. Statin therapy is contraindicated in patients with pregnancy.

## Antiplatelet therapy

T2DM is associated with increased blood thrombogenicity among patients with non-ST elevation acute coronary syndrome [110]. Platelet P2Y_12_ expression is increased fourfold in patients with T2DM, and platelet activation and hypercoagulation in T2DM induce a prothrombotic state and result in an increased risk for CVD events [111].

### Aspirin

The use of aspirin decreases the risk of T2DM among healthy men but not among women [112]. Low-dose aspirin (81 or 100 mg) is neutral for CVD outcomes and increases the risk for gastrointestinal bleeding when used for primary prevention among patients with T2DM [113]. Contemporary meta-analyses show that the use of aspirin for the primary prevention of CV events needs to be reconsidered [114, 115] due to inconsistent CVD benefits and an increased bleeding risk.

In secondary prevention trials, meta-analyses showed that medium-dose aspirin (75–325 mg/day) reduces vascular events over 2 years [116], an aspirin dose of 75–150 mg daily is a protective regimen for patients with occlusive vascular events [117], and aspirin therapy for the secondary prevention of CVD has long been established [118]. Nonetheless, the longest duration of the included trials was just 4 years, and the aspirin effect for longer term use this requires caution, particularly in the post-acute setting [119]. Meta-analyses showed that early aspirin discontinuation in patients with acute coronary syndrome or percutaneous coronary intervention (PCI) prevented bleeding events with a neutral effect on CVD outcomes [120], and the duration of aspirin use (3 months) in patients undergoing complex PCI reduced the risk of bleeding without increasing the risk of ischemic events [121]. In acute settings, reducing the duration of aspirin use (< 3 months) for secondary prevention of CVD may be proposed.

### *P2Y*_*12*_inhibitors

Compared with aspirin, P2Y_12_ inhibitors reduce the risk of myocardial infarction and stroke in secondary prevention [122]; thus, P2Y_12_ inhibitors may be a useful option for secondary prevention. P2Y_12_ inhibitors include prasugrel, ticagrelor and clopidogrel. Long-term administration of clopidogrel reduces the risk of myocardial infarction or vascular death, and the overall efficacy is superior to that of aspirin [123]. The benefits of prasugrel and ticagrelor over clopidogrel are even greater, and a reduction in CVD mortality was observed only with ticagrelor [124]. Ticagrelor exerts similar or greater inhibition of platelet reactivity than prasugrel in diabetic patients with CAD [125]. For patients after PCI, discontinuation of aspirin within 1 to 3 months with continued P2Y_12_ inhibitor monotherapy has a neutral effect on MACE outcomes with a reduction in bleeding [121].

### Rivaroxaban

Rivaroxaban has been shown to reduce the incidence of CVD and an increased risk of bleeding among patients with chronic coronary syndrome [126]. Compared to warfarin, rivaroxaban reduces stroke, myocardial infarction and MACEs with a lower risk of bleeding among patients with atrial fibrillation and diabetes [127].

### Treatment choice

Depending on risk stratification and the risk of bleeding, ESC guidelines recommend that aspirin be considered for primary prevention among patients with a risk of CVD or a diagnosis of CVD and low risk of bleeding [41, 94].

Intensive secondary prevention is indicated for patients with T2DM and CVD. Aspirin therapy (75–162 mg/day) is recommended as a secondary preventive strategy for patients with T2DM and atherosclerotic CVD. For diabetic patients with an aspirin allergy, clopidogrel (75 mg/day) should be used [128]. Long-term dual antiplatelet therapy is approved for patients with additional high-risk markers.

The addition of clopidogrel to aspirin for people with CVD risk or established CVD is associated with a reduction in myocardial infarction and ischemic stroke, however it also leads to an increase in bleeding [129]. This regimen may increase CVD death among patients with DM and microalbuminuria (≥ 30 μg/mL) [130]. The CV benefit of clopidogrel plus aspirin is reduced in T2DM due to high platelet reactivity, and increasing the dose of clopidogrel and aspirin may enhance antiplatelet effects [131, 132]. The benefit of an intensive antiplatelet regimen in these patients is still unclear. The National Institute for Health and Care Excellence (NICE) recommends prasugrel plus aspirin for people with ST elevation myocardial infarction after PCI. Prasugrel or ticagrelor plus aspirin is recommended for people with non-ST elevation myocardial infarction after PCI. Clopidogrel and oral anticoagulants other than prasugrel or ticagrelor for up to one year are recommended for people with acute coronary syndrome and atrial fibrillation after PCI [133]. The recommended duration of these regimens is > 30 months [134], while meta-analyses have indicated inconsistent results on the duration of dual antiplatelet therapy after PCI with drug-eluting stents [135, 136], and discontinuation of aspirin within 1 to 3 months with continued P2Y_12_ inhibitor monotherapy is recommended [137].

For patients with DM and atrial fibrillation or peripheral artery disease, ESC guidelines recommend rivaroxaban therapy [94]. Rivaroxaban plus aspirin is the preferred long-term antithrombotic regimen for patients with chronic coronary syndrome and high-risk factors [126].

## Conclusion

The current main targets for the control of glycemia, lipids and blood pressure levels in patients according to the most commonly used guidelines should be included as an individualized strategy to prevent CVD in T2DM (Tables 2, 6 and 7). Although the incidence and mortality rate of T2DM-related CVD have decreased, the prevalence and mortality rate of CVDs in patients with T2DM continues to rise, and most T2DM-related CVDs may be prevented by lifestyle modification and the use of adjunctive drugs. The notion of T2DM-related CVD care has transitioned from comprehensive medical intervention to precision diabetes therapy. For T2DM patients with established CVD, the GLP-1 agonists, SGLT2 inhibitors, and blood-pressure and lipid-lowering drugs provide an improved precision treatment approach.

## Data Availability

Not applicable.

## Abbreviations

T2DM: Type 2 diabetes mellitus
CVD: Cardiovascular disease
HF: Heart failure
CAD: Coronary artery disease
HFpEF: Heart failure with preserved ejection fraction
HFrEF: Heart failure with reduced ejection fraction
HbA1c: Hemoglobin A1c
SGLT2: Sodium-glucose cotransporter 2
GLP-1: Glucagon-like peptide-1 receptor
DPP4: Dipeptidylpeptidase-4
RCT: Randomized controlled trial
AHA: American Heart Association
ADA: American Diabetes Association
ESC: European Society of Cardiology
EASD: European Association for the Study of Diabetes
GFR: Glomerular filtration rate
pre-DM: Prediabetes
ACE: Angiotensin-converting enzyme
ARB: Angiotensin II receptor blockers
TGs: Triglycerides
LDL-C: Low-density lipoprotein cholesterol
HDL-C: High-density lipoprotein cholesterol
PCSK9: Proprotein convertase subtilisin/kexin 9
PCI: Percutaneous coronary intervention
MACE: Major adverse cardiac event
NICE: National Institute for Health and Care Excellence
